# Assessing preparedness of non-hospital health centers to provide primary emergency care: a systematic literature review

**Published:** 2019-08

**Authors:** Mehrdad Amir Behghadami, Ali Janati, Homayoun Sadeghi-Bazargani, Masoumeh Gholizadeh, Farzad Rahmani, Morteza Arab-Zozani

**Affiliations:** ^*a*^Iranian Center of Excellence in Health Management (IceHM), Department of Health Service Management, School of Health Services Management and Medical Informatics, Tabriz University of Medical Sciences, Tabriz, Iran.; ^*b*^Department of Statistics and Epidemiology, Faculty of Health, Tabriz University of Medical Sciences, Tabriz, Iran.; ^*c*^Emergency Medicine Department, Sina Medical Research and Training Hospital, Tabriz University of Medical Sciences, Tabriz, Iran.; ^*d*^Iranian Center of Excellence in Health Management (IceHM), School of Management and Medical Informatics, Tabriz University of Medical Sciences, Tabriz, Iran

## Abstract

**Background:**

Preparedness of non-hospital health centers, particularly in remote areas, plays a vital role in saving the patients who are severely injured. In addition, it is compatible with the overall purpose of developing health systems to improve capability of the system to be responsive to the needs of society. However, the point is if these centers are prepared enough to provide emergency care or not. This study aimed to identify prevalent domains related to the concept of assessing preparedness of non-hospital centers to provide primary emergency care in order to develop a conceptual framework through a systematic literature review.

**Methods:**

Five databases including PubMed, Scopus, Web of science, Barakat Knowledge Network Systems (BKNS) and Scientific Information Database (SID) were searched in English and/or Persian languages with no time limit until March, 2018. Manual search and grey literature were also done. According to the eligibility criteria, all the studies were independently tracked by two researchers. Studies were appraised using the Mixed Method Appraisal Tool (MMAT). Identified studies that met the inclusion criteria were synthesized using a directed content analysis method.

**Results:**

Out of 3014 studies, 15 studies were included for data synthesis. The synthesis of literature resulted in the emergence of 13 domains and 25 sub-domains. Then, they were categorized based on Donabedian’s triple model and a conceptual framework was developed. In this framework, 6 domains were put in input, 6 in processes, and 1 domain in outcome. Of the 15 included studies, 1 study considered 10 domains and 14 other studies considered 4 to 8 domains out of 13 synthesized domains. The most prevalent synthesized domains were “medical supplies and equipment” and “human resources”, which were considered in 15 studies.

**Conclusions:**

In this systematic review, a conceptual framework was constructed that identifies elements that significantly affect the preparedness of these centers. This framework may assist managers to take a comprehensive approach to assess these centers.

**Keywords:**

Non-hospital Health centers, Primary emergency care, Preparedness, Assess

